# Left atrial strain determinants and clinical features according to the heart failure stages. New insight from EACVI MASCOT registry

**DOI:** 10.1007/s10554-022-02669-x

**Published:** 2022-07-01

**Authors:** Giovanni Benfari, Giulia Elena Mandoli, Julien Magne, Marcelo Haertel Miglioranza, Roberta Ancona, Vlatka Reskovic Luksic, Maria Concetta Pastore, Ciro Santoro, Blazej Michalski, Alessandro Malagoli, Denisa Muraru, Erwan Donal, Bernard Cosyns, Thor Edvardsen, Bogdan Alexandru Popescu, Matteo Cameli, MASCOT investigators

**Affiliations:** 1 Section of Cardiology, Department of Medicine, University of Verona, Piazzale A. Stefani 1, 37126 Verona, Italy; 2 Department of Medical Biotechnologies, Division of Cardiology, University of Siena, Siena, Italy; 3 CHU Limoges, Hôpital Dupuytren, Service Cardiologie, 87042 Limoges, France; 4 INSERM U1094, University of Limoges, CHU Limoges, IRD, U1094, GEIST, 2, rue Marcland, 87000 Limoges, France; 5 Institute of Cardiology/University Foundation of Cardiology, Porto Alegre, Brazil; 6 UOC Cardiologia/UTIC – “Santa Maria della” Grazie Hospital Pozzuoli, Pozzuoli, Italy; 7 Department of Cardiovascular Diseases, University Hospital Centre Zagreb, Zagreb, Croatia; 8 Department of Advanced Biomedical Sciences, Federico II, University Hospital, Naples, Italy; 9 Department of Cardiology, Medical University of Lodz, Lodz, Poland; 10 Division of Cardiology, Nephro-Cardiovascular Department, Baggiovara Hospital, University of Modena and Reggio Emilia, Modena, Italy; 11 Department of Medicine and Surgery, University of Milano-Bicocca, Milan, Italy; 12 University of Rennes, CHU Rennes, Inserm, LTSI – UMR 1099, 35000 Rennes, France; 13 Centre for Cardiovascular Diseases, University Hospital of Brussels, Brussels, Belgium; 14 Department of Cardiology, Center for Cardiological Innovation, Oslo University Hospital, Rikshospitalet, Oslo, Norway; 15 University of Oslo, Oslo, Norway; 16 Department of Cardiology, University of Medicine and Pharmacy “Carol Davila” – Euroecolab, Emergency Institute for Cardiovascular Diseases “Prof. Dr. C. C. Iliescu”, Sos. Fundeni 258, 022328 Bucharest, Romania

**Keywords:** Atrial function, Heart failure, Echocardiography

## Abstract

Few studies analyzed left atrial (LA) peak atrial longitudinal strain (PALS) determinants, particularly across heart failure (HF) stages. We aimed to analyze the pathophysiological and clinical PALS correlates in a large multicentric prospective study. This is a multicenter prospective observational study enrolling 745 patients with HF stages. Data included PALS and left ventricular global longitudinal strain (LV-GLS). Exclusion criteria were: valvular prosthesis; atrial fibrillation; cardiac transplantation; poor acoustic window. Median global PALS was 17% [24–32]. 29% of patients were in HF-stage 0/A, 35% in stage-B, and 36% in stage-C. Together with age, the echocardiographic determinants of PALS were LA volume and LV-GLS (overall model R^2^ = 0.50, p < 0.0001). LV-GLS had the strongest association with PALS at multivariable analysis (beta: −3.60 ± 0.20, p < 0.0001). Among HF stages, LV-GLS remained the most important PALS predictor (p < 0.0001) whereas age was only associated with PALS in lower HF-stage 0/A or B (R = − 0.26 p < 0.0001, R = − 0.23 p = 0.0001). LA volume increased its association to PALS moving from stage 0/A (R = − 0.11; P = 0.1) to C (R = − 0.42; P < 0.0001). PALS was the single most potent echocardiographic parameter in predicting the HF stage (AUC for B vs. 0/A 0.81, and AUC vs. 0/A for C 0.76). PALS remained independently associated with HF stages after adjusting for ejection fraction, E/e′ ratio, and mitral regurgitation grade (p < 0.0001). Although influenced by LV-GLS and LA size across HF stages, PALS is incrementally and independently associated with clinical status. LA function may reflect a substantial part of the hemodynamic consequences of ventricular dysfunction.

**Supplementary Information:**

The online version contains supplementary material available at 10.1007/s10554-022-02669-x.

## Introduction

Left atrial (LA) peak longitudinal strain (PALS) can be easily obtained by speckle tracking echocardiography (STE) and reliably reflects the LA phasic function [1]. PALS has demonstrated association with major cardiovascular outcomes in several clinical settings including heart failure (HF) [2–5]. However, only a few studies on healthy individuals have analyzed PALS pathophysiological determinants [6–8], and no evidence on the spectrum of HF stages is available.

The LA function mirrors the elevation of left ventricular (LV) filling pressure [9], is influenced by LV performance [10], and can be a buffer for volume overload as in the case of functional mitral regurgitation [11, 12]. These factors are frequently present in various combinations in HF and may generate complex pathophysiological interactions. Furthermore, whether PALS determinants are similar in all the HF stages has never been explored, leaving doubts on its clinical value’s universality in patients with HF.

The present study aims to analyze PALS pathophysiological and clinical correlates in a large multicentric prospective study, including comprehensively characterized HF patients from stage 0 to C.

## Methods

### Study cohort

From July to October 2018, the european association of cardiovascular imaging (EACVI) heart imagers of tomorrow (HIT) Members and/or Ambassadors experienced in echocardiography, who agreed to participate, were asked to collect clinical and echocardiographic data in various HF stages. The complete protocol of the original study is reported elsewhere [13]. Briefly, the images were acquired using machines from a single vendor (GE Medical Systems, Milwaukee, WI). HF stages were defined according to recommendations as Stage-A, for patients at risk for HF but without current or prior symptoms or signs of HF and structural or biomarkers evidence of heart disease. Stage-B, for patients without current or prior symptoms or signs of HF, but evidence of structural heart disease or abnormal cardiac function, or elevated natriuretic peptide levels. HF Stage-C, for patients with current or prior symptoms and/or signs of HF caused by a structural and/or functional cardiac abnormality [14]. We also included patients without CV risk factors (i.e., healthy subjects) labeled as stage 0. Exclusion criteria were: unfeasible measurements of both LA and LV strain, valvular prosthesis; atrial fibrillation; cardiac transplantation; poor acoustic window (but not sub-optimal). Institutional review board approval was obtained for the study in each center, and all subjects signed the informed consent. All procedures were conducted in accordance with the Declaration of Helsinki.

### Standard echocardiography and clinical variables

A complete clinical evaluation was performed at the time of the enrollment including a comprehensive cardiovascular risk factor assessment and evaluation of major comorbidities. Each echocardiogram was performed using a high-quality machine equipped with a 1.5- and 3.6-MHz transducer. The LV and LA volumes were assessed from apical four- and two-chamber views using the biplane modified Simpson’s rule, according to current recommendations [15]. The LV mass was assessed from 2D images using the truncated ellipsoid technique and subsequently indexed for body surface area. The diastolic function was assessed using pulsed Doppler and Tissue Doppler Imaging according to recommendations [16]. Mitral, aortic, and tricuspid valve disease were evaluated and graded using an integrated multiparametric approach [16].

### Speckle tracking echocardiography

**Fig. 1:**
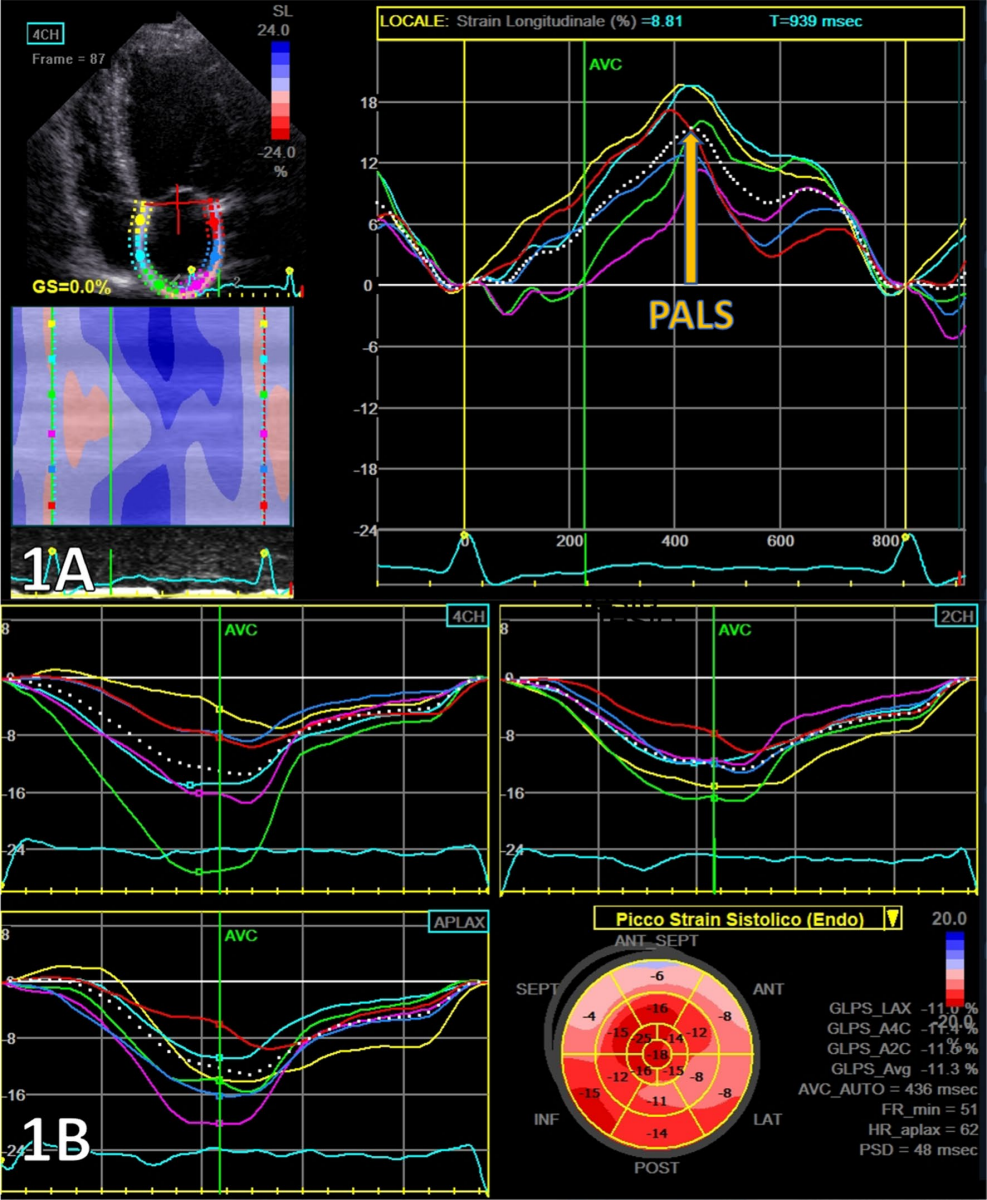
Representative example of two-dimensional STE–derived PALS measurements **A** and LV GLS **B** from an apical four-chamber view

A frame rate of 40–80 fps was required for the STE measurements, and images were analyzed by semi-automatic software (EchoPAC, GE, USA). Two different echocardiographers, blinded to each other, performed the measurements in each center. A predefined approach was adopted by all centers for LA strain measurements [17]. PALS was obtained from apical four- and two-chamber views and then averaged (Fig. 1A).

LV strain quantification was assessed by an endocardial point-and-click approach, the ROI was manually defined and eventually modified, in each apical view (four-, two- and three-chambers). A total of 18 segments were obtained, six for each view, and GLS reported (Fig. 1B). Advanced measurements were performed by an expert physician and a cardiologist in training, blinded from each other measures.

### Statistical analysis

Patients were divided into three groups according to HF stages. The normality of the distribution was assessed using the Shapiro Wilk test. Data are summarized as means ± SD, median and interquartile range (for continuous variables), or numbers and percentages (for qualitative variables) as appropriate. Comparisons across patient groups were performed using analysis of variance (ANOVA followed by the post hoc Bonferroni test for multiple comparisons), Wilcoxon test, or Chi-squared test. PALS determinants were selected according to their univariable association with PALS and physiological plausibility. Subsequently, to identify the parameters with an independent association to PALS, a multiple linear regression analysis was performed (using PALS as a continuous dependent variable) as well as logistic regression analysis (using PALS above vs. below the median cohort’s value). To further validate the results, a neural network (66% training, 33% validation) model was applied.

PALS clinical significance was tested with multinomial logistic regression analysis using HF stages (0 and A, B, C) or NYHA class (I vs. ≥ II) as the dependent variables; the area under the curve (AUC) is reported. All analyses were performed using JMP®, Version 14. SAS Institute Inc., Cary, NC. The significance level was set at 0.05 for all analyses.

## Results

### Characteristics of the study cohort

A total of 745 with complete clinical and echocardiographic evaluation formed the study cohort. Clinical and echocardiographic characteristics are presented in Table 1. The median age was 63 [50–72] years, 58% male. There was a balanced proportion of HF stages: stage 0/A in 29% (n = 214), stage B in 35% (n = 263), and stage C in 36% (n = 268). Despite similar sex distribution and body surface area, multiple clinical and echocardiographic characteristics differ significantly across the HF stages. In particular, PALS values were significantly lower from stage 0/A to stage C (p < 0.0001) as shown in the Supplementary Fig. 1.

**Table 1:** Clinical and echocardiographic characteristics across HF stages

Clinical variables	Overall (n = 745)	HF stage 0/A (n = 214; 29%)	HF stage B (n = 263; 35%)	HF stage C (n = 268; 36%)	p value
Age, y	63 [50–72]	47.5 [34.8–61]	64 [54–72]	70 [61–78]	< 0.0001
Men, n (%)	434 [58]	117 [55]	158 [60]	159 [60]	0.4
BSA, m^2^	1.84 ± 0.20	1.83 ± 0.20	1.86 ± 0.19	1.84 ± 0.22	0.3
Diabetes, n (%)	139 [19]	15 [17]	42 [20]	82 [31]	< 0.0001
Dyslipidemia, n (%)	226 [30]	27 [13]	80 [30]	119 [44]	< 0.0001
HT, n (%)	360 [48]	44 [21]	142 [54]	174 [65]	< 0.0001
Heart rate (bpm)	69 ± 12	69 ± 11	68 ± 10	70 ± 13	0.07
SBP (mmHg)	130 [118–140]	123 [112–132]	130 [120–140]	130 [118–141]	< 0.0001
DBP (mmHg)	80 [70–86]	79 [70–84]	80 [70–89]	80 [70–87]	0.3
Echocardiography					
RWT	40 [33–49]	37 [33–42]	41 [35–48]	44 [34–50]	< 0.0001
IVS (mm)	11 [9–12]	9 [8–10]	11 [10–12]	12 [10–13]	< 0.0001
PW (mm)	10 [9–11]	9 [8–10]	10 [9–11]	11 [9–12]	< 0.0001
LV mass index (g/m^2)^	97 [76–122]	74 [62–85]	103 [88–123]	118 [94–140]	< 0.0001
EDV index (mL/m^2^)	54 [45–67]	51 [43–58]	55 [44–68]	60 [48–79]	< 0.0001
ESV index (mL/m^2^)	23 [18–31]	19 [16–23]	24 [19–31]	28 [19–43]	< 0.0001
LV-EF (%)	58 [50–64]	61 [57–66]	57 [49–62]	53 [40–61]	< 0.0001
LAVI max bipl (mL/m^2^)	32 [25–42]	24 [21–30]	33 [26–41]	41 [33–55]	< 0.0001
E/A ratio	1.09 [0.76–1.58]	1.24 [0.98–1.66]	0.92 [0.71–1.35]	1.00 [0.71–1.76]	< 0.0001
E/e′ ratio	9.2 [6.9–13]	7.04 [5.84–8.31]	9.2 [6.9–11.5]	12.9 [9.1–17.4]	< 0.0001
Mod/severe AS	116 (16)	0 (0)	45 (17)	71 (26)	< 0.0001
Mod/severe MR	118 (16)	0 (0)	40 (15)	78 (29)	< 0.0001
PALS (%)	17 [14–32]	33 [26–40]	24 [19–31]	18 [12–24]	< 0.0001
LV-GLS (%)	–18 [20–15]	–20 [21–18]	–18 [20–15]	–16 [19–12]	< 0.0001

Data are expressed as median [IQR] or mean ± DS

*AS* aortic stenosis; *BSA* body surface area; *DBP* diastolic blood pressure; *EDV* end-diastolic volume; *EF* ejection fraction; *ESV* end-systolic volume; *GLS* global longitudinal strain; *HF* heart failure; *HT* hypertension; *IVS* inter ventricular septum; *LA* left atrial; *LAVI* left atrial volume index; *LV* left ventricular; *MR* mitral regurgitation; *PALS* peak atrial longitudinal strain; *PW* pulsed wave; *RWT* relative wall thickness; *SBP* systolic blood pressure

### Determinants of PALS in the whole cohort

**Fig. 2:**
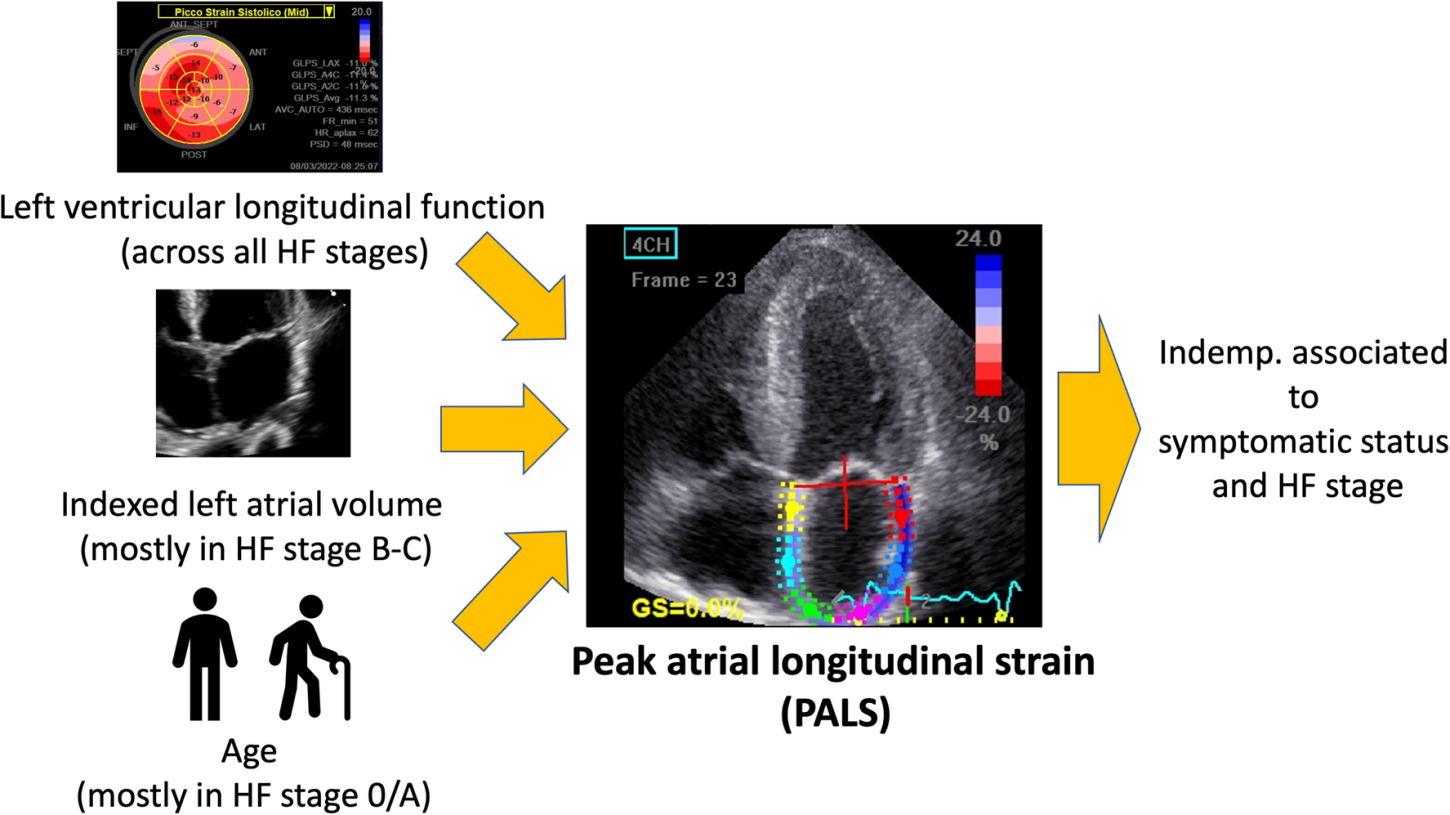
The figure summaries the most important PALS determinants

The major echocardiographic features associated with PALS were age (R = − 0.38, p < 0.0001), LAVI (R = − 0.53, p < 0.0001), and LV GLS (R = − 0.56, p < 0.0001) (Fig. 2). Univariable and multivariable regression analysis are presented in Table 2 for PALS as a continuous variable (overall multivariable model R^2^ = 0.50, p < 0.0001) as well as for PALS above the cohort median value, using a neural network to validate the prediction provided similar results (R^2^ = 0.53 for the patients in the training cohort and R^2^ = 0.52 for the validation cohort).

**Table 2:** Univariable and multivariable multiple regression model for PALS associated features in the whole cohort and according to HF-stages.

	PALS determinants		Determinants of reduced PALS (below the median cohort value)	
Whole cohort	Univariable Beta ± SE	Multivariable Beta ± SE	Univariable OR (95%CI)	Multivariable OR (95%CI)
Age, per 5 year	− 1.25 ± 0.11; p < 0.0001	− 0.71 ± 0.09; p < 0.0001	1.28 (1.22–1.36); p < 0.0001	1.23 (1.15–1.31); p < 0.0001
LAVI, per 3 mL/m^2^	− 0.96 ± 0.07; p < 0.0001	− 0.63 ± 0.06; p < 0.0001	1.24 (1.19–1.29); p < 0.0001	1.20 (1.14–1.25) p < 0.0001
LV-GLS, per 3%	− 4.28 ± 0.23; p < 0.0001	− 3.6 ± 0.20; p < 0.0001	2.26 (1.96–2.23); p < 0.0001	2.39 (2.01–2.85) p < 0.0001
HF stage 0/A				
Age, per 5 year	− 0.95 ± 0.22; p < 0.0001	− 0.61 ± 0.20; p = 0.03	1.33 (1.18–1.51); p < 0.0001	1.29 (1.13–1.49); p = 0.0003
LAVI, per 3 mL/m^2^	− 0.81 ± 0.33; p = 0.01	− 0.78 ± 0.28; p = 0.002	1.05 (0.89–1.23); p = 0.5	1.04 (0.86–1.25) p = 0.7
LV-GLS, per 3%	− 5.46 ± 0.68; p < 0.0001	− 5.01 ± 0.67; p = 0.007	3.70 (2.26–3.06); p < 0.0001	3.48 (2.07–5.85) p < 0.0001
HF stage B				
Age, per 5 year	− 0.76 ± 0.17; p < 0.0001	− 0.56 ± 0.15; p = 0.0003	1.18 (1.08–1.30) p < 0.0001	1.17 (1.06–1.30); p = 0.001
LAVI, per 3 mL/m^2^	− 0.59 ± 0.11; p < 0.0001	− 0.63 ± 0.09; p < 0.0001	1.14 (1.07–1.21) p < 0.0001	1.19 (1.10–1.28) p < 0.0001
LV-GLS, per 3%	− 2.76 ± 0.37; p < 0.0001	− 3.02 ± 0.36; p < 0.0001	1.85 (1.46–2.34) p < 0.0001	2.29 (1.73–3.02) p < 0.0001
HF stage C				
Age, per 5 year	− 0.16 ± 0.20; p = 0.4	− 0.14 ± 0.15; p = 0.3	1.06 (0.95–1.17); p = 0.2	1.14 (1.01–1.29); p = 0.03
LAVI, per 3 mL/m^2^	− 0.51 ± 0.08; p < 0.0001	− 0.44 ± 0.07; p < 0.0001	1.18 (1.09–1.26); p < 0.0001	1.17 (1.09–1.26) p < 0.0001
LV-GLS, per 3%	− 2.86 ± 0.27; p < 0.0001	− 2.74 ± 0.26; p < 0.0001	1.69 (1.37–2.10); p < 0.0001	1.93 (1.48–2.51) p < 0.0001

PALS is used as dependent variable in the model either as continuous variable (left part of the table) or as categorical (right part of the table)

*HF* heart failure; *GLS* global longitudinal strain; *LAVI* left atrial volume index; *LV* left ventricular; *OR* odds ratio; *PALS* peak atrial longitudinal strain

At the univariable analysis, E/e′ was significantly associated with PALS (R =  −0.46, p < 0.0001). However, adding E/e′ to the other determinants in a nested regression model did not significantly increase the PALS prediction (R^2^ grew from 0.50 to 0.51). Therefore E/e′ was not included in the final model. Interestingly, no meaningful association was noted between PALS and diastolic blood pressure or heart rate; a complete list of PALS echocardiographic correlates is presented in Supplementary Table 1. Repeating the analysis using the advanced echocardiographic measurements did not affect the results (multivariable PALS prediction R^2^ was 0.52, p < 0.0001).

**Fig. 3:**
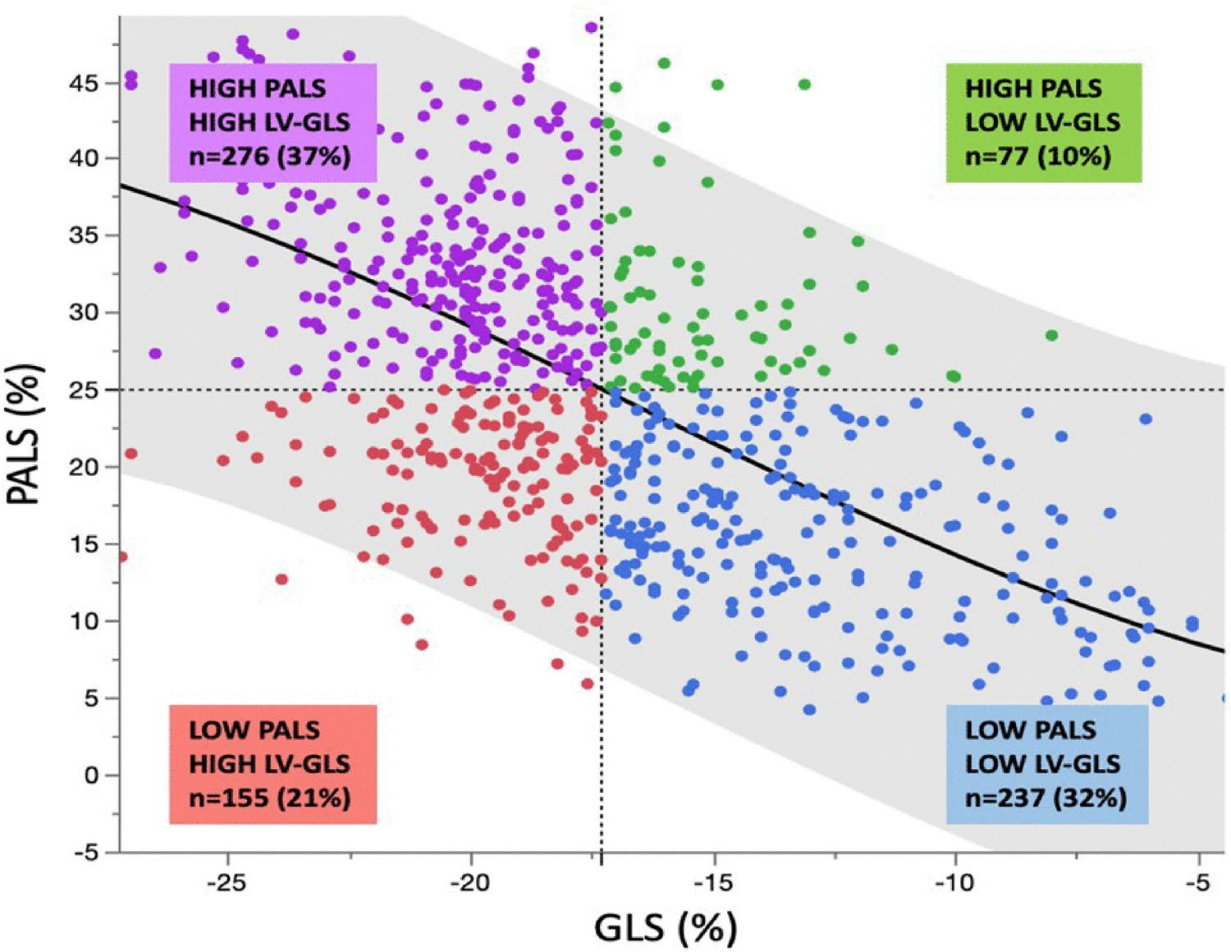
Scatterplot illustrating the relationship between PALS and GLS. Colors identify four patients group based on the cohort’s median PALS and GLS. The green dots (preserved PALS and reduced GLS) are by far the less represented and crowded towards the fitting line

The LV GLS showed the strongest correlation with PALS, as illustrated in Fig. 3: the cohort was also divided into four groups based on median PALS (≤ vs. > 25%) and GLS (≤ vs. > 18%). Concordance (preserved PALS and GLS or reduced PALS and GLS) was present in 69% of cases. In the remaining (31%) so-called discrepant cases, reduced PALS in the presence of GLS above the median was the predominant presentation (21% of cases), and only 10% of the cohort presented preserved PALS and reduced GLS with values clustered towards the fitting line.

### Determinants of PALS according to HF stages

**Fig. 4:**
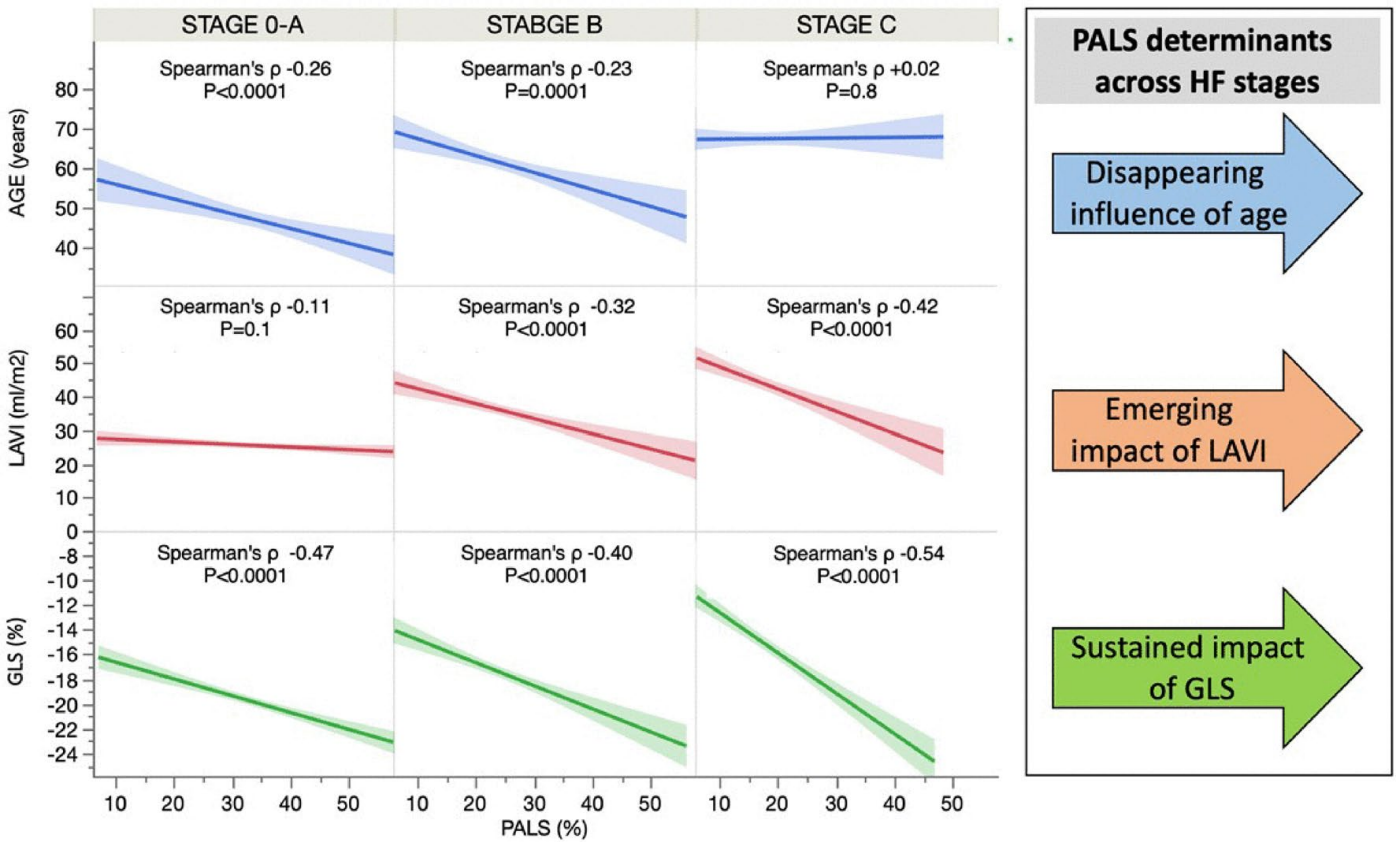
Summary of the main PALS associated echocardiographic features and their interaction with H stage. Notably, as highlighted in the right panel, the age and LA volume effect on PALS is highly influenced by the HF stage, whereas GLS association with PALS remains stable in all HF stage

The relative importance of each PALS determinant was analyzed according to the HF stages as illustrated in Fig. 4. LV GLS remains steadily the most critical predictor in any stage; age was particularly important in stage 0/A and showed no role in HF stage C (p for interaction = 0.03). Conversely, LAVI showed a modest relationship with PALS in stage 0/A and gained more importance moving to stage B and C. Again, adding E/e′ increased the model prediction minimally only in stage C (R^2^ grew from 0.39 to 0.41). Among the 268 patients in HF stage C, 67 (25%) had ischemic etiology, 85 (32%) related to systemic hypertension, 67 (25%) to valvular heart disease, and the remaining 49 (18%) were labeled as non-ischemic or mixed etiology. Interestingly, there was no interaction between the HF etiology and the PALS-LV GLS relationship (p for interaction = 0.6).

### Clinical consequences of reduced PALS

Multiple echocardiographic features characterized the HF stages (Table 1, right columns). PALS was independently associated with HF stages (< 0.0001) when tested together with its determinants. Furthermore, it was the single most powerful echocardiographic parameter in predicting the HF stage (AUC for stage B vs. 0/A = 0.81, and AUC for stage C vs. 0/A = 0.76). The PALS remains independently associated with HF stages after adjusting for ejection fraction, E/e′, mitral regurgitation grade (p < 0.0001). Results did not change, adding any of the other echocardiographic HF features (data not showed).

**Fig. 5:**
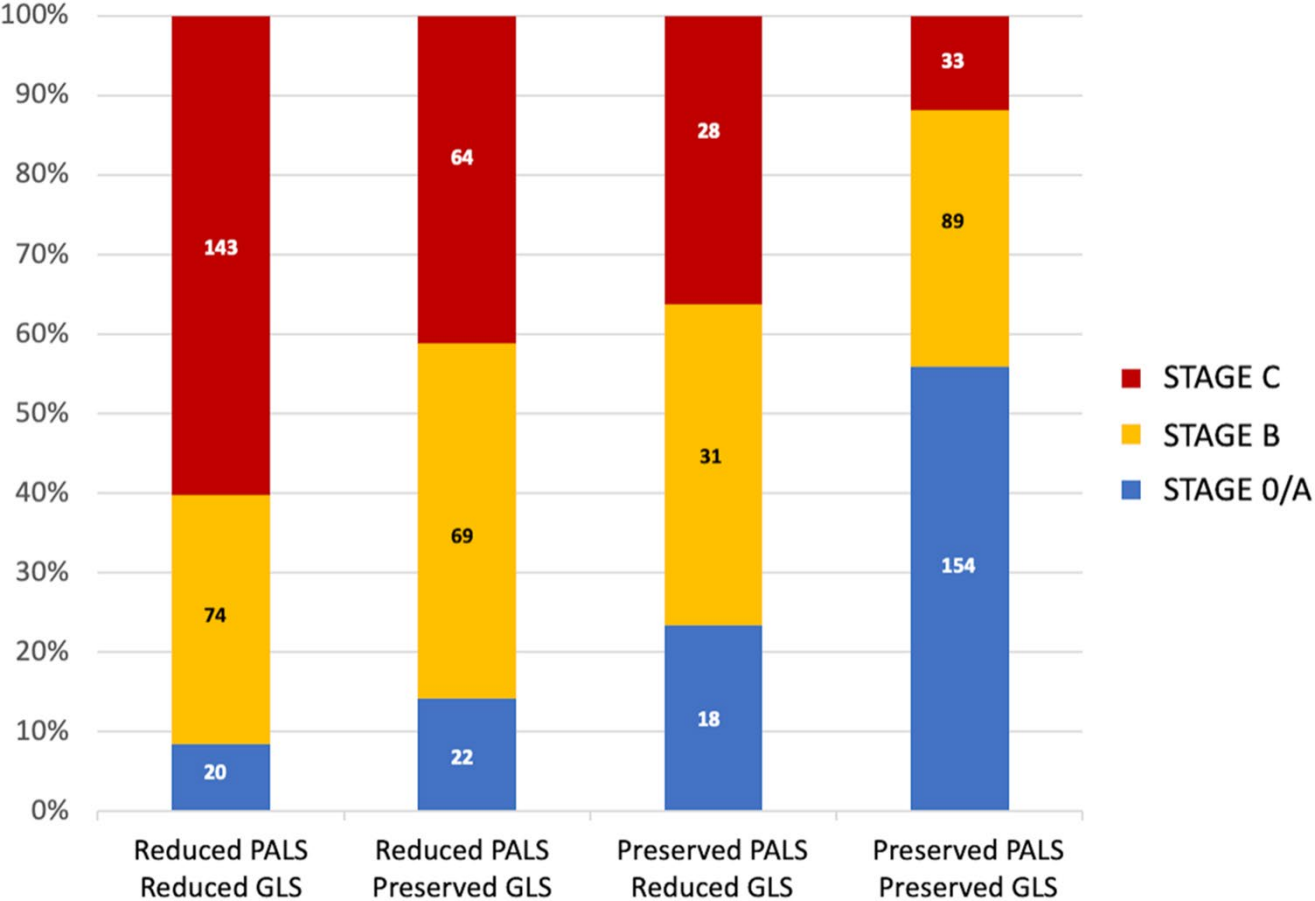
Proportion of PALS/GLS grouping across HF stages. The absolute number of patients in each subgroup is reported within the columns

Figure. 5 shows the four cohort subgroups based on median PALS and GLS. There is an increasing proportion of patients with more severe HF stages moving from patients with higher to lower PALS. Of note, reduced PALS was associated with slightly more severe HF vs. reduced GLS (middle columns).

A total of 268 (36%) patients were symptomatic (NYHA class ≥ 2) at the time of echocardiographic examination. PALS was significantly associated with symptomatic status in a multivariable model including his determinants (OR for PALS 3 unit increase 0.88 [0.81–0.96], p = 0.003), and even after adjustment for LV ejection fraction, mitral regurgitation grade and E/e′ ratio (OR for PALS 3 unit increase 0.87 [0.81–0.95], p = 0.003).

## Discussion

The present study, taking advantage of a unique large multicentric prospective cohort, demonstrates that:

- The PALS value is mostly influenced by age, LA size, and LV GLS.
- The LV GLS is the strongest determinant of PALS; of note concomitant preserved PALS and reduced LV GLS is rare.
- PALS is related to age among patients with HF stage 0/A or B, whereas the LA size is more important in more advanced HF stages (B and C).
- Despite its multiple determinants, PALS is the superior echocardiographic parameter for characterization of the HF stages and for predicting the presence of symptoms.

### Clinical relevance of atrial function in HF

Growing evidence is accumulating on the clinical importance of the LA function—mostly measured by PALS—in all HF stages, particularly stages A and B which are characterized by early LV and LA remodeling. Indeed, LA mechanics reflect the degree of functional and morphological chamber adaptation better than the conventional echocardiographic parameters. As a paradigmatic example, patients with hypertension and diabetes have shown an early reduction of all LA myocardial deformation components even in the presence of normal LA size [18]. Furthermore, the LA function has a tight relationship with the patient clinical status, the LV filling pressure, and the pulmonary pressure [19, 20]. Lastly, the LA function has provided incremental predictive value vs. conventional parameters for almost all major HF outcomes [4, 11].

### PALS and GLS across HF stages

The present study is one of the largest cohorts, specifically addressing PALS correlates, and even more important the first study to analyze differences in atrial function across multiple HF stages. There are a multitude of physiological features associated with PALS [4], but only a few are closely associated with PALS., The relationship between PALS and LV GLS has been previously reported in smaller studies conducted on healthy volunteers [6, 21], confirming the role of PALS as a marker of subclinical LV dysfunction [22]. Thus, an accumulation of evidence suggests that PALS may help identify asymptomatic patients at higher risk of events in the lower HF stages. This pathophysiological role is less understood in the more advanced HF stage, where the interaction between atrial function and other hemodynamic features increases in complexity.

The present study’s relatively large sample size and the wide range of explored atrial and ventricular strain values enlighten details of the PALS-GLS relationship. Indeed, the association between the two advanced echocardiographic measurements is not strictly linear, and it is rare to present with reduced GLS but preserved PALS. This aspect agrees with the potential anticipatory nature of reduced PALS towards a GLS reduction and reveals that low GLS almost invariably relates to low PALS. In other words, in patients with relatively preserved LV GLS, PALS value mostly reflects intrinsic atrial properties and holds its distinctive pathohistological role [22]; with a progressive reduction in GLS, the PALS value seems to depend more and more on LV function rather than on atrial properties.

Another novel result of the present study is that HF-stage influences the age-PALS relationship. Age seems a significant determinant of atrial stiffness in patients with no or limited cardiac involvement; however, in more advanced HF stages (B and C), different hemodynamic or biochemical drives increase LA stiffness, which becomes age-independent in HF-stage C. It is to be acknowledged that patients in HF stage B and C are older than those in stage 0/A, but the age-PALS correlation progressively decreases with the HF stage despite the similar age in stages B and C. This supports the change in determinants rather than the age ranges as a possible explanation for the significant interaction.

LA volume showed an independent association with PALS, which increased in strength moving from HF stage 0/A to C. The explanations for this behavior reside in the multiple pathophysiological meaning of LA size, which reflects the elevation of diastolic filling pressure over time [23, 24], the burden of mitral regurgitation [25], it is a sensitive morphophysiological expression of the severity of LV dysfunction, and to be a useful global index of the cardiovascular risk [26]. Despite this strong physiological background, overall, PALS values were only partially explained by the LA volume (R = 0.46), making plausible the incremental value of the atrial function over LA size demonstrated in a variety of clinical contexts [27–29].

### Limitations

The lack of follow-up is the main limitation of the study; however, the present study provides contemporary insights into PALS’ clinical meaning. Indeed, this atrial parameter was the most characterizing echocardiographic parameter of HF-stage, which is mostly a clinically defined stage, and it is not based on LA chamber characteristics or any of the PALS determinants. In invasive studies conducted in advanced HF, LA strain was the best determinant of pulmonary capillary wedge pressure among the echocardiographic features [30]. This was confirmed across patient groups with varying LV ejection fraction [31], which ultimately explains the incremental clinical value (for symptoms as well as HF stage identification) observed in our study. There is a pathophysiological explanation of this PALS clinical value over its most important determinants: the atrial function may buffer the increased ventricular filling pressure as well as volume overload due to functional mitral regurgitation, therefore being the ultimate determinant of the patients’ symptomatic status [32]. This has been demonstrated for PALS in the context of mitral regurgitation [20]. Another limitation of the present study is the lack of a core lab for the assessment of echocardiographic measurements. However, no interaction between the primary regression model and centers was seen. This, together with the stable results using measurements performed by a consultant or those performed by a trainee enhance the applicability in the real world of our results.

## Conclusion

In this prospective large multicenter study LV function by GLS, LA size, and age are independently associated with PALS with hierarchical importance influenced by patients’ HF stage. LA strain is a crucial HF stage characterizing feature and strongly relates to the presence of symptoms.

## Supplementary Information

Below is the link to the electronic supplementary material.

Supplementary file1 (DOCX 914 kb)

## Data Availability

Data will be made available upon reasonable request.
