# Supplementary material for: Left atrial strain determinants and clinical features according to the heart failure stages. New insight from EACVI MASCOT registry
Source: Int J Cardiovasc Imaging. 2022 Jul 1;38(12):2635–44. doi: 10.1007/s10554-022-02669-x (PMC9708811; doi:10.1007/s10554-022-02669-x)

**Supplementary table 1:** PALS echocardiographic correlates.

|  | **Correlation coefficient (r) ; p-value** | **Number of patients with available data** |
| --- | --- | --- |
| Age | -0.38; <0.0001 | n=744 |
| Body surface area | -0.11; 0.003 | n=744 |
| Body Mass index | -0.27; <0.0001 | n=744 |
| Heart rate | -0.06; 0.1 | n=743 |
| Systolic Blood Pressure | -0.11; 0.002 | n=733 |
| Diastolic Blood Pressure | 0.09; 0.02 | n=733 |
| End-diastolic volume | 0.18; <0.0001 | n=742 |
| Left Ventricular Ejection Fraction | +0.35; <0.0001 | n=742 |
| Global longitudinal strain | -0.56; <0.0001 | n=745 |
| Left ventricular Mass index | -0.47; <0.0001 | n=742 |
| Relative wall thickness | -0.11; 0.003 | n=744 |
| Left atrial Volume index | -0.53; <0.0001 | n=742 |
| E | -0.07; 0.05 | n=744 |
| E/A | -0.01; 0.7 | n=742 |
| E/e’ | -0.46; <0.0001 | n=697 |

**Supplementary Figure 1.** Box and whiskers plot illustrating the PALS value across heart failure stages.


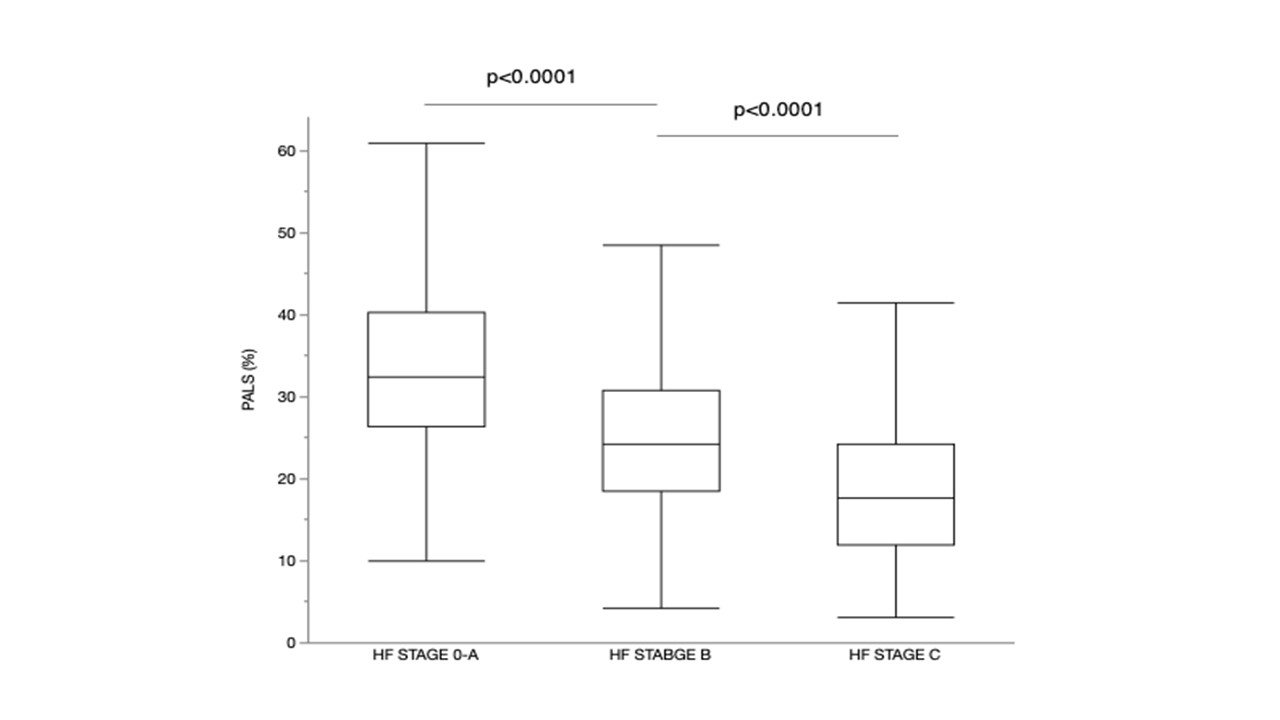

Supplement: Supplementary file 1 — Supplementary file1 (DOCX 914 kb) [file 10554_2022_2669_MOESM1_ESM.docx]
